# Features of Two Cases with 18q Deletion Syndrome

**DOI:** 10.4274/Jcrpe.1183

**Published:** 2014-03-05

**Authors:** Elif Özsu, Gül Yeşiltepe Mutlu, Ayşegül Büte Yüksel, Şükrü Hatun

**Affiliations:** 1 Kocaeli University Medical Faculty, Department of Pediatric Endocrinology and Diabetes, Kocaeli, Turkey

**Keywords:** 18q deletion, MC4R, Autoimmunity

## Abstract

The 18q Deletion syndrome is seen in 1 out of 10 000 live births. The main features of the syndrome are short stature, hearing loss, hypotonia, mental retardation, endocrine disorders and autoimmunity. Here, we present 2 patients with this syndrome admitted to our clinic who were found to have insulin resistance in addition to mental retardation, short stature, autoimmune thyroiditis and hearing loss. The need to perform a karyogram analysis in cases presenting with these features is emphasized.

## INTRODUCTION

The incidence of 18q Deletion syndrome is reported as 1 in 10 000 live births. The characteristic features of the syndrome are short stature, hearing loss, hypotonia, mental retardation and endocrine disorders, accompanied by autoimmunity (1).

We report two patients with this syndrome, admitted to our clinic with various symptoms including insulin resistance (IR) in addition to mental retardation, short stature, autoimmune thyroiditis and hearing loss. The aim of this report is to underline the phenotypic features and endocrine disorders typical for this syndrome and to emphasize the need for karyograms in patients with these findings.

## CASE REPORT 1

A female patient aged 12 years and 3 months was referred to the pediatric endocrinology outpatient clinic from the cardiology department for her short stature. Birth was by normal vaginal delivery and was difficult but did not require the hospitalization of the infant. Birth weight was 2500 g. She was reported to have acquired head control by the 3^rd^ month of life but was noted to be retarded in her neuromotor development at age 6 months and was able to walk at age 2.5 years. Hearing impairment was identified when she was 7 years old. Based on her Denver Development Test results which indicated defective neuromotor development, it was recommended that she receive special education. At age 11 years, she had been diagnosed to have aortic stenosis and was being followed by the cardiology department. Her two siblings and her non-consanguineous parents were healthy. The patient’s weight was 41 kg [-0.8 standard deviation score (SDS)], height 133.5 cm (-3.5 SDS) and head circumference was 50 cm.

Bone age was 11 years. Midparental height (MPH) was 151.8 cm (3-10p). At physical examination, abdominal obesity and scoliosis of the lumbar vertebrae were noted. Inspection also revealed a thin upper lip, prognathism and ear auricle anomaly. The 4^th^ and 5^th^ metacarpals of both hands were short and overriding of the toes was present. The thyroid gland was nonpalpable. Pubertal status was compatible with stage 3. A systolic murmur was heard in all heart focuses. Bilateral hearing loss and right nystagmus were also present. Laboratory examination results showed that serum lipid values were in the upper normal range (total cholesterol: 176 mg/dL, triglyceride: 187 mg/dL) and that her thyroid stimulating hormone (TSH) level was high (8.16 IU/mL). Her anti-thyroid peroxidase (anti-TPO) level was 83.9 IU/mL and her anti-thyroglobulin (anti-TG) level was 92.6 IU/mL. Thyroid imaging revealed a thyroid volume of 7.3 mL. The patient was diagnosed to have Hashimoto thyroiditis and subclinical hypothyroidism and was started on L-thyroxine (L-T4). Immunoglobulin A (IgA) values were measured and found to be normal. Insulin-like growth factor-1 (IGF-1) and IGF binding protein-3 (IGFBP-3) levels were low (<-2 SD) for age.

At follow-up, annual height growth rate was 6.5 cm/year. Weight gain was also found to be accelerated. At 13 years and 8 months, acanthosis nigricans appeared on the neck and armpits and striae in the lumbar regions. In a review of her history, no laboratory data suggesting iatrogenic Cushing’s disease were found, but the patient had received local steroid treatment for atopic eczema for approximately 3 years.

Metformin therapy was started following determination of a peak insulin level of 400 IU/mL. Oral glucose tolerance test (OGTT) was performed and homeostatic model assessment of IR (HOMA-IR) was measured at 5.7. The patient lost weight and IR regressed with treatment (HOMA-IR: 2.43).

Chromosomal structure in our patient was deletion of 18 (q21.31-q23). Genes with deletion were identified as RAX, LMAN1, TNFRSF11A, BCL2, MC4R, FVT1 and CTDP1 using comparative genomic hybridization (CGH).

## CASE REPORT 2

A female patient aged 13 years and 3 months was referred to the pediatric endocrinology outpatient clinic from the allergy department due to her being overweight.

The patient’s birth history was normal. She was born by normal vaginal delivery, with a birth weight of 3500 g. Increased weight was noted to develop at age 5 or 6. The patient was also reported to have mental retardation and retardation in motor development. She walked at age 3 and talked around age 5-6 years. The patient was under psychiatric observation for her mental retardation and behavioral problems and had been given Risperdal. She also was reported to have allergic asthma from birth. The parents were second-degree relatives. Two siblings (a girl and a boy) were alive and healthy.

The patient’s weight was 61 kg (1.8 SDS) and her height was 140 cm (-2.68 SDS). Her bone age was 12 years. Body mass index was 30, compatible with her proportionate short stature.

Physical examination revealed a patient with a short neck, a broad nasal bridge, a downturned mouth and prognathism. Marked acanthosis nigricans was observed on her neck. Shortness of the 4^th^ and 5^th^ metacarpals and cubitus valgus were also present. Puberty was compatible with stage 5.

Thyroid function test results [TSH: 798 IU/mL, free T4 (fT4): 0.160 ng/dL, anti-TPO: 800 IU/mL and anti-TG: 869 IU/mL] were compatible with autoimmune thyroiditis. Due to high TSH values, a pituitary MR was performed and adenoma was ruled out. Ultrasonographic evaluation results were also in line with a diagnosis of thyroiditis (thyroid volume: 8.7 mL). Dyslipidemia (triglycerides: 575 mg/dL, total cholesterol: 337 mg/dL) and hyperinsulinism with a peak value of 130 mU/mL measured during OGTT were the other pathologies identified. IgA values were measured and found to be normal. IGF-1 and IGFBP-3 levels were low (<-2 SD) for age.

Urinary ultrasound revealed a horseshoe kidney. Cardiologic examination was normal. Auditory tests revealed bilateral hearing loss.

Peripheral blood karyotype analysis of this patient also revealed deletion in the regions of 21.3-23, belonging to the long arms of the 18^th^ chromosome.

**Figure 1:**
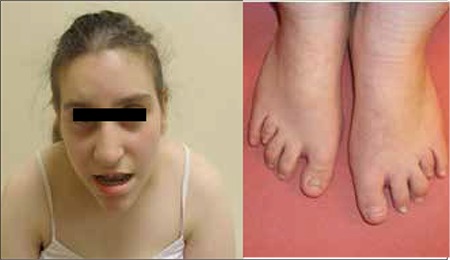
Facial and foot appearance of Case 1

**Figure 2:**
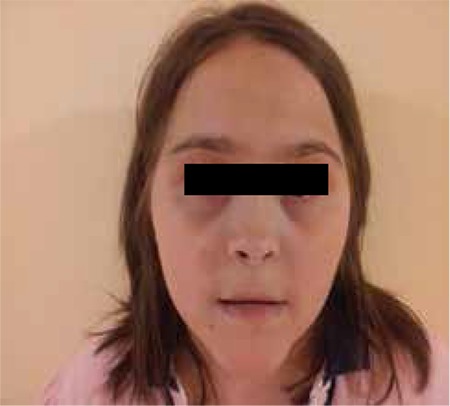
Facial appearance of Case 2

**Figure 3:**
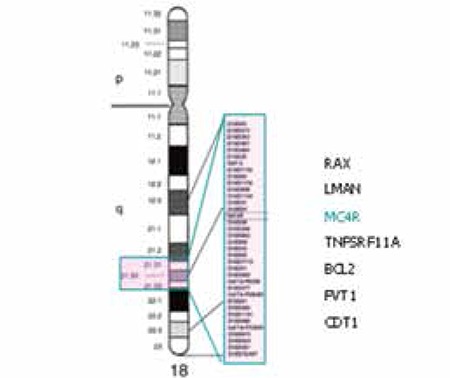
Chromosome 18

**Figure 4:**
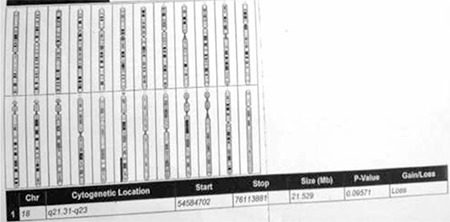
Image of a comparative genomic hybridization (CGH)

Clinical and laboratory data on the two patients are given in Tables 1 and 2. Facial appearance of the two patients is demonstrated in Figures 1 and 2. Results of genetic analysis are presented in Figures 3 and 4.

## DISCUSSION

The most common findings of 18q Deletion syndrome, which has a wide phenotype, are a characteristic facial appearance (microcephaly, palatal defects, a carp-shaped mouth, a short palpebral fissure and outer ear abnormalities), mental retardation, short stature, hypotonia, hearing loss and feet deformities (1,2). Deletion of 18q22.3 is the chromosomal abnormality in which typical clinical findings are most commonly seen.

All components generally reported in the literature were present in our cases. Nystagmus, strabismus, optic atrophy and retinal degeneration have been reported in addition to auricular findings and hearing defects (1). Optic atrophy and nystagmus were present in our Case 1. The reported incidence of cardiac defects is approximately 24%; of these, atrial and ventricular septal defects, absent pulmonary valve, pulmonary stenosis and aortic valve defects are the most common (3). While Case 1 was being followed by the cardiology department with a diagnosis of bicuspid aortic valve and valvular aortic stenosis, cardiological examination of Case 2 was normal. Gunes et al (4) reported mitral insufficiency in a case with an 18q21-qter karyogram. 

External ear anomalies and hearing loss are also among the major components of the syndrome (1). Auricular anomaly and a sensorineural hearing loss of 50% were found in Case 1 and a hearing loss of 40% with no auricular anomaly was found in Case 2.

One of the most important findings in these cases is short stature; a height SDS below -2 is reported in 64% of these patients and annual height growth rates are below average values in 70% of the cases. Approximately 70% of the cases show an inadequate growth hormone (GH) response in stimulation tests (5).

Height percentiles were below -2.5 SDS in both our cases. Annual growth rate in Case 1 was more than 5 cm/year until menarche and 2 cm/year thereafter. Case 2 was referred to us when she was at Tanner stage 5, after which annual growth rate was 1 cm/year. Unfortunately, we were not able to perform GH stimulation tests in our two patients, but IGF-1 and IGFBP-3 levels in both cases were below -2 SDS for chronological and bone age. Since height velocity appeared to be near normal in Case 1 and could be estimated only after a late stage of puberty in Case 2, we can hypothesize that the short stature in both cases was probably the result of poor growth in early childhood, possibly due to inadequate GH secretion.

Susceptibility to autoimmune diseases is significantly lower in 18q Deletion syndrome compared to the normal population. An association has been reported between the 18q21 region and diseases such as Hashimoto, Graves, rheumatoid arthritis and type 1 diabetes. Autoimmunity-related IgA deficiency has also been observed. According to some authors, deletions in the 18q chromosome affect critical genetic loci, resulting in activated autoimmunity. On the other hand, no immunoglobulin genes are encoded on the 18th chromosome and IgA deficiency has also been reported in other 18th chromosomal abnormalities (ring chromosome 18 and 18q Deletion syndrome) as well as from 18q Deletion syndrome.

IgA deficiency is seen approximately in 24% of cases but not in all patients with activated autoimmunity (6). The IgA levels of our patients were normal.

While a moderately high TSH level was detected in Case 1 during routine laboratory tests for short stature, TSH and autoantibody levels in Case 2, admitted for obesity and short stature, were very high. Autoimmune thyroiditis in our cases indicated active autoimmunity and also suggested an allergic affinity. Autoimmune hyperthyroidism has also been rarely reported (7).

Obesity has been reported in cases with 18q12.2;21.1 deletion and this may be related to the amount of deleted genes (8). Patients with this karyogram have a different facial appearance to the classical one and are often obese. This relationship suggests a genotype and phenotype correlation in 18q Deletion syndrome.

Fenestra et al (9) analyzed the chromosomal structure of 29 syndromic cases using the CGH technique. They determined six cases with proximal deletion, 22 with terminal deletion and aberration between different chromosomes in one patient. The syndrome has been found to be associated with microcephaly (18q21.33), short stature (18q12.1-q12.3 18q21.1-q21.33, 18q22.3-q23), delayed myelination (18q22.3-q23), GH deficiency (18q22.3-q23) and congenital aural atresia (18q22.3). The main critical point for 18q Deletion syndrome has been identified as the 4.3Mb 18q22.3-q23 region (9).

In a study of 151 cases, Cody identified the region which determines phenotype as 18q22.3-q23. Five genes regulating demyelination and response to GH, four genes dealing with renal malformation and three genes related to aural atresia were detected in this region (10).

The chromosomal structure of our patients was 18(q21.31-q23). Array CGH identified the genes with deletion as RAX, LMAN1, TNFRSF11A, BCL2, MC4R, FVT1 and CTDP1. While the loss of the MC4R gene is a probable cause of obesity in the affected cases (11), the presence of horseshoe kidney in our Case 2 may also be associated with this loss (Figure 3).

In conclusion, this report on two rare cases emphasizes the importance of karyogram analysis in cases with more than one endocrine pathology accompanied by hearing loss and mental retardation.

## Figures and Tables

**Table 1:**
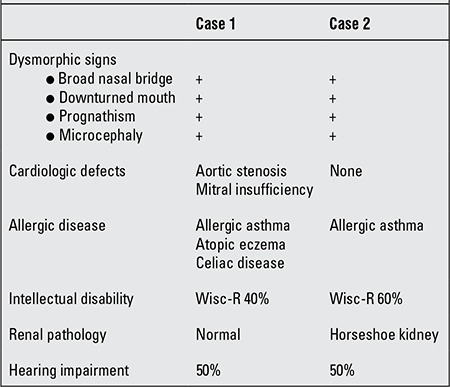
Clinical findings in our two patients

**Table 2:**
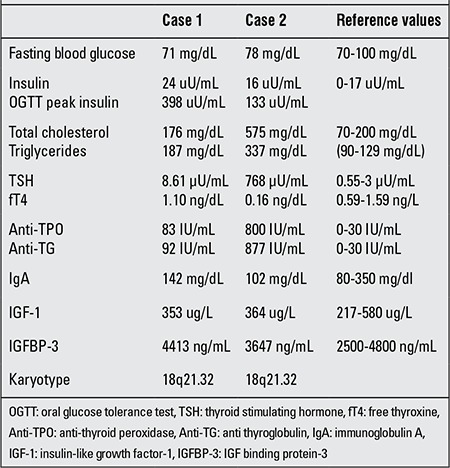
Laboratory findings in our two patients
